# Supplementary material for: Atomic-Scale Friction on Monovacancy-Defective Graphene and Single-Layer Molybdenum-Disulfide by Numerical Analysis
Source: Nanomaterials (Basel). 2020 Jan 2;10(1):87. doi: 10.3390/nano10010087 (PMC7023280; doi:10.3390/nano10010087)
Supplement: Supplementary file 1 [file nanomaterials-10-00087-s001.pdf]

Supplementary Materials

Atomic-Scale Friction on Monovacancy-Defective Graphene and Single-Layer Molybdenum-Disulfide by Numerical Analysis

Haosheng Pang [1,†], Hongfa Wang [1,†], Minglin Li [1,2,*] and Chenghui Gao [1,*]

[1] School of Mechanical Engineering and Automation, Fuzhou University, Fuzhou, Fujian 350002, China; m150210010@fzu.edu.cn (H.P.); hongfawang05@163.com (H.W.)
[2] Fujian Key Laboratory of Medical Instrumentation and Pharmaceutical Technology, Fuzhou University, Fuzhou, Fujian 350002, China
* Correspondence: liminglin@fzu.edu.cn (M.L.); gch@fzu.edu.cn (C.G.)
† These authors contributed equally to this work.

Table 1. The parameters for the LJ potential V_{LJ}.

LJ Potential	E_0 (eV)	r_0 (nm)	cutoff radius (nm)
T-Mo	0.001052	0.3452	0.86300
T-S	0.007360	0.3943	0.98575
T-C	0.002840	0.3400	1.19000

T means the carbon atom of the single-atom tip, and C means the carbon atoms in the graphene. S and Mo mean the sulfur and molybdenum atoms of $SLMoS_2$, respectively. These parameters have been demonstrated in previous works [1,2].

Table 2. The parameters for the long-range interaction V_{long} of Figure 2(a) and (b).

Sample	*E* (eV)	b_1 (nm)	b_2(nm)	*c* (nm)	*d* (nm)
Graphene	0.09	0.12	0.14	0.04	0.49
$SLMoS_2$	2.50	0.10	0.12	0.53	6.08

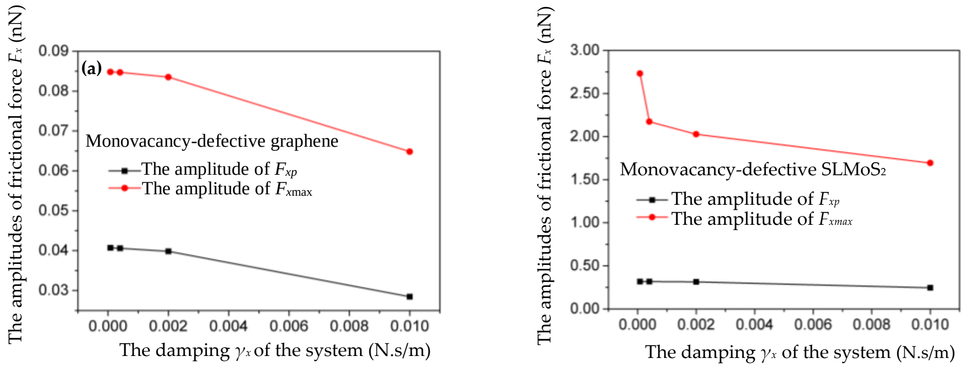

Figure S1. The amplitudes of the maximum frictional forces F_{xmax} and the periodic frictional forces F_{xp} of the monovacancy-defective graphene (a) and $SLMoS_2$ (b) with different dampings γ_x of the system (γ_x = $8*10^{-5}$ N.s/m, $4*10^{-4}$ N.s/m, $2*10^{-3}$ N.s/m and 10^{-2} N.s/m).

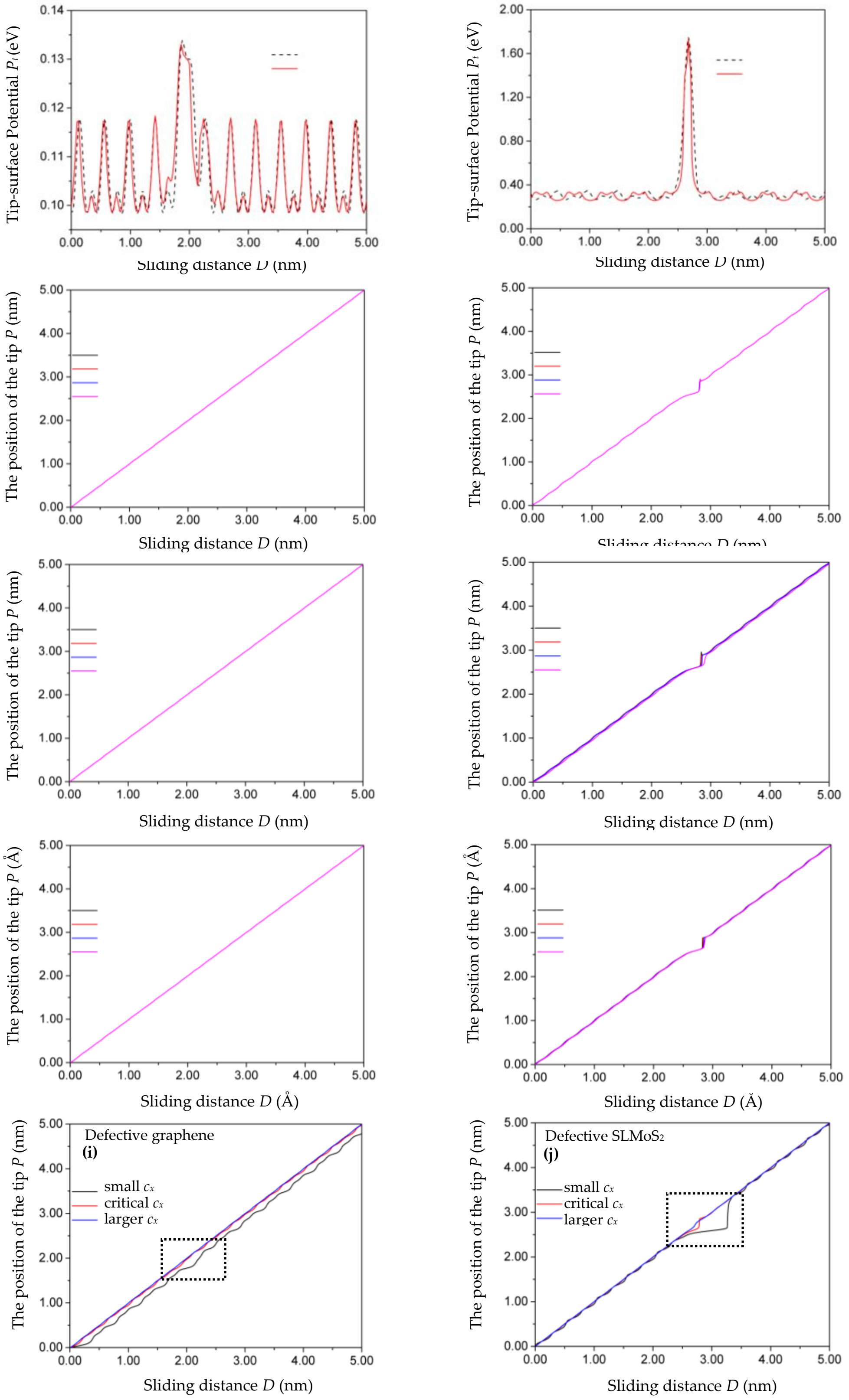

Figure S2. The tip-surface potential *Pt* vs scanning distance *D* curves for sliding paths along AC orientation of the monovacancy-defective graphene (a) and $SLMoS_2$ (b). The solid red curves and the dashed black curves in (a) and (b) were obtained by calculating the LJ potential (V_{LJ}) and by fitting the LJ potential (V_{LJ}) via the modified interaction potential (V_{total}), respectively. The position of the single-atom

tip P vs the scanning distance D curves for sliding paths along AC orientation of the monovacancy-defective graphene ((c), (e), (g) and (i)) and $SLMoS_2$ ((d), (f), (h) and (j)) with different effective mass of the system m_x, damping of the system γ_x, sliding velocity V_m and spring constant of the pulling spring c_x. The critical spring constants c_x of the monovacancy-defective graphene (i) and $SLMoS_2$ (j) are the curvatures of the tip-surface interaction (V_{total}'') at the vacancy defect site of defective graphene and $SLMoS_2$ (V''_{carbon} = 1.42 for the monovacancy-defective graphene and V''_{SLMoS2} = 14.76 for the monovacancy-defective $SLMoS_2$). The small and larger c_x are based on their respective critical values c_x and separated by an amplitude of 5 times. Specifically, the small, critical and larger c_x of the monovacancy-defective graphene in Figure S2 (i) is 0.28 N/m, 1.42 N/m and 7.10 N/m, respectively. The small, critical and larger c_x of the monovacancy-defective $SLMoS_2$ in Figure S2 (j) is 2.95 N/m, 14.76 N/m and 73.8 N/m, respectively. The dotted box in Figure S2(i) and (j) represent the the stick-slip behaviors in the vicinity of the vacancy defect of the monovacancy-defective graphene and $SLMoS_2$, respectively.

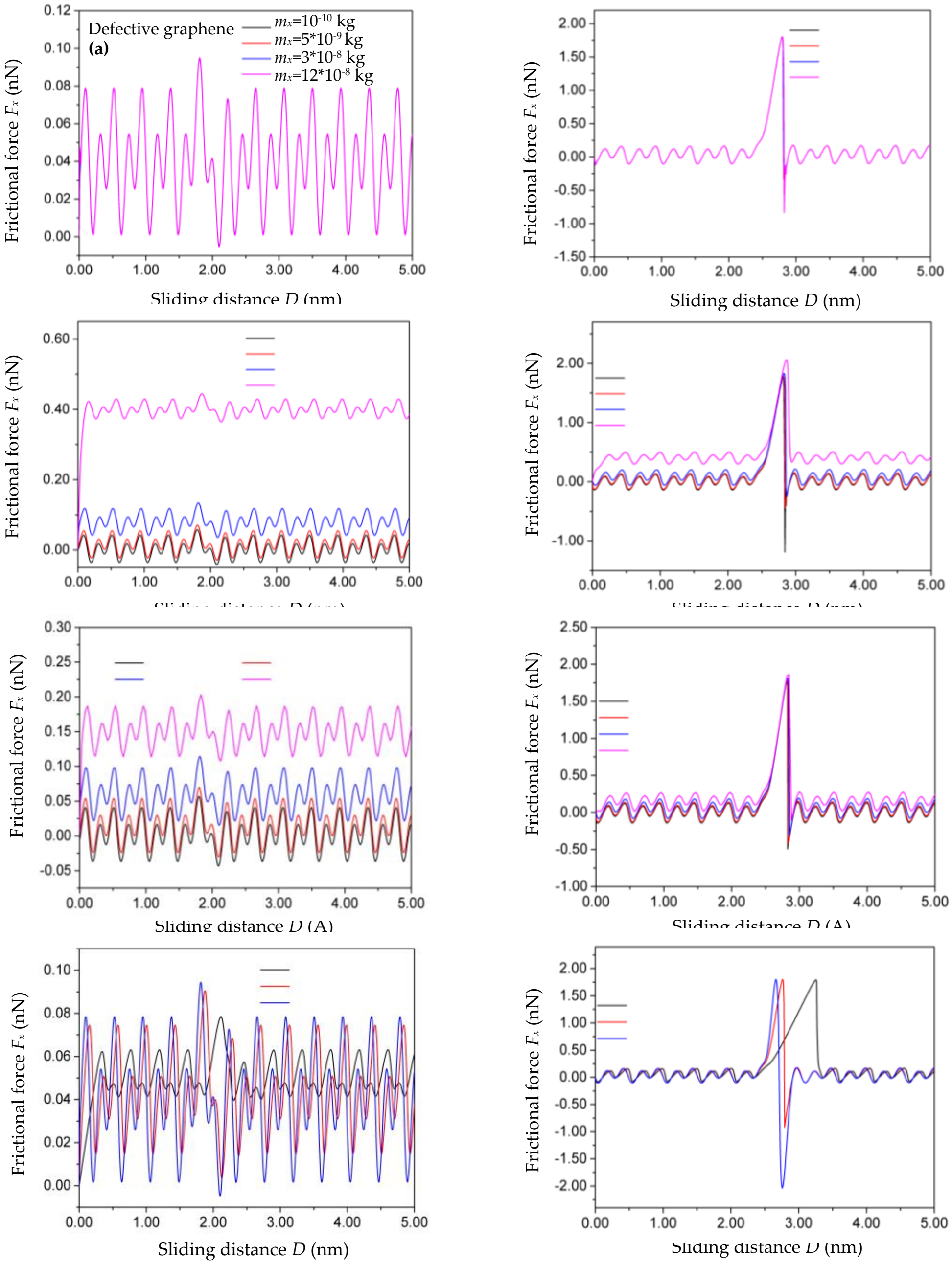

Figure S3. The frictional force F_x vs the sliding distance D curves for sliding paths along AC orientation of the monovacancy-defective graphene ((a), (c), (e) and (g)) and SLMoS2 ((b), (d), (f) and (h)) with different effective mass of the system m_x, damping of the system γ_x, sliding velocity V_m and spring constant of the pulling spring c_x.

References:

1. Sun, X.Y.; Wu, R.N.; Xia, R.; Chu, X.H.; Xu, Y.J. Effects of Stone-Wales and vacancy defects in atomic-scale friction on defective graphite. *Applied Physics Letters* **2014**, *104*, 2605.
2. Li, M.; Zhuo, W.; Pang, H.; Lai, L. Improving the atomic-resolution AFM imaging of monolayer MoS_2 for worn tips: a molecular dynamics study. *Japanese Journal of Applied*

Physics **2019**, *58*, 055003.
